# Seasonality in mood and behaviours of Japanese residents in high-latitude regions: transnational cross-sectional study

**DOI:** 10.1186/s13030-016-0084-2

**Published:** 2016-12-05

**Authors:** Yumiko Kurata, Shuhei Izawa, Shinobu Nomura

**Affiliations:** 1 Advanced Research Center for Human Sciences, Waseda University, 2-579-15, Mikajima, Tokorozawa, Saitama 359-1192 Japan; 2 Occupational Stress Research Group, National Institute of Occupational Safety and Health, 6-21-1 Nagao, Tama-ku, Kawasaki-shi, Kanagawa 214-8585 Japan; 3 Faculty of Human Sciences, Waseda University, 2-579-15, Mikajima, Tokorozawa, Saitama 359-1192 Japan

**Keywords:** Depression, Japanese resident, Migration, Seasonal affective disorder, Seasonal changes

## Abstract

**Background:**

Daylight hours in high-latitude regions tend to be longer than those in Japan in summer, and shorter than those in Japan in winter. For example, daylight hours in London in winter are one-third those of Tokyo. Therefore, this study investigated and compared seasonal changes in mood and behaviours of Japanese individuals living in and outside Japan.

**Methods:**

Surveys were conducted with Japanese residents in summer and winter in the UK (*n* = 106), Nordic countries (*n* = 40), Southeast Asia (*n* = 50), and Japan (*n* = 96). First, summer and winter General Health Questionnaire–28 (GHQ28) scores of each regional group were analysed. Subsequently, month-wise differences in mood and behaviours were compared across the four geographical regions.

**Results:**

Summer and winter GHQ28 scores of participants living in the UK and Nordic countries differed significantly, while no seasonal differences were observed for residents in Japan and Southeast Asia. Further, in the UK and Nordic countries, summer was associated with better mood and more activity, while winter was linked to lowered mood and reduced activity.

**Conclusion:**

The results indicate that Japanese living in the UK and Nordic countries (high-latitude regions) experience seasonal fluctuations in depressive symptoms that may be linked to drastic seasonal environmental changes. Observed over a 12-month period, their mood and behaviour declined in winter and improved in summer. Therefore, considering the prevalence of overseas stressors that differ from those in their home country, it is necessary to investigate the effectiveness of support systems that help migrants adapt to seasonal changes in high-latitude regions.

## Background

With increasing Japanese migration, prevalence rates of mental disorders and suicides among migrants have increased. According to the annual report by the Ministry of Foreign Affairs of Japan, both the number of Japanese people living overseas on a long-term basis, and the prevalence of mental disorders, mental illness, and suicide of such people have increased [1, 2]. Approximately 300,000 Japanese individuals live in areas that lie above latitude 50°N and experience considerable seasonal changes. Summers are marked by more daylight hours, while winters have fewer daylight hours. For example, the total daylight hours in the United Kingdom (UK) during the winter (December to February) are one-third of those in Tokyo. Migration to higher latitude areas is associated with higher prevalence of seasonal affective disorder (SAD), especially among non-indigenous populations [3–6]. An analysis of seasonal changes in individuals’ mood, emotion, sleep, and activity levels according to four regions of differing latitudes in Japan showed that fluctuations were significantly larger in the high-latitude region [7]. Compared to the United States (US) and Europe, seasonal changes in individuals’ behaviours in Japan are less frequent and have a different profile [8]. Among Asian, White, and Black individuals in the UK, Asians were the only ethnic group that showed significant seasonal variations in depression, with a greater number of depressive episodes in winter [9]. Seasonal depression peaked in winter for three groups—Asian, Asian-British, and White—with the highest incidence of winter depression recorded for Asians [10]. Further, gender and ethnicity may be major risk factors for low mood in winter [9].

We expected that seasonal changes would have a substantial impact on Japanese residing in high-latitude regions. Existing research has demonstrated that seasonal changes in mood and behaviour among individuals living in different regions in Japan are associated with latitudinal differences; however, this phenomenon has not been sufficiently investigated among Japanese living overseas [7].

In this two-part study, the effects of seasonal changes were investigated in the first analysis while differences between geographical regions were examined in the second analysis. Therefore, a longitudinal study comparing health scores in two seasons—summer and winter—was first conducted to determine the effect of seasonal changes on depression among Japanese living abroad and in the Kanto region, Japan. The scores of Japanese residing in and outside Japan on the Japanese versions of the General Health Questionnaire 28-Japanese version (GHQ28) [11] and the Global Seasonality Score-Japanese version (GSS) [7] were compared. Second, seasonal fluctuations in mood and behaviour were examined over a 12-month period based on the degree of seasonal dependence, as determined by GSS scores.

## Methods

### Participants

Participants were recruited from the members of the Japanese Resident Association or Japanese communities in the UK (*n* = 105), Nordic countries (Finland, Sweden, and Norway: *n* = 40), and Southeast Asia (Singapore and Malaysia; *n* = 51). Ninety-six Japanese participants were residents of the Tokyo area (*n* = 96). They were all aged 20 years or older (Table 1).

**Table 1:** Characteristics of participants

	*n*	Mean age (*SD*)	Sex	Mean age	*SD*	Duration of stay (months)	Occupation	Family status	Children	Seasonality score (GSS)
UK	105	41.1 (±8.10)	Male *n* = 23	40.83	9.51	90	Student 4 Housewife 45 Full-time 41 Part-time 7 Other 8	Unmarried 18 Married 80 Others 7	Yes 64 No 41	Low: *n* = 66 (62.9%) Moderate: *n* = 15 (14.3%) High: *n* = 24 (22.9%)
			Female *n* = 82	41.18	7.73					
Nordic countries	40	39.08 (±10.06)	Male *n* = 6	30	5.87	99.67	Student 6 Housewife 10 Full-time 15 Part-time 4 Other 5	Unmarried 9 Married 31	Yes 18 No 22	Low: *n* = 32 (80%) Moderate: *n* = 1 (2.5%) High: *n* = 7 (17.5%)
			Female *n* = 34	40.68	9.83					
Southeast Asia	51	40.82 (±5.95)	Male *n* = 17	40.24	7.27	56.27	Student 1 Housewife 27 Full-time 16 Part-time 4 Other 2	Unmarried 3 Married 47 Other 1	Yes 42 No 8 No answer 1	Low: *n* = 46 (90.2%) Moderate: *n* = 0 (0%) High: *n* = 5 (9.8%)
			Female *n* = 34	41.06	5.28					
Japan	96	43.97 (12.68)	Male *n* = 24	47.25	12.07		Student 10 Housewife 27 Full-time 37 Part-time 10 Other 12	Unmarried 30 Married 64 Others 2	Yes 48 No 48	Low: n = 72 (75.0%) Moderate: *n* = 12 (12.5%) High: *n* = 12 (12.5%)
			Female *n* = 72	43.04	12.78					

*GSS* Global Seasonality Score. Based on participants’ GSS scores, respondents were divided into low (˂8), moderate (8–10), and high (≥11) seasonality groups

**Fig. 1:**
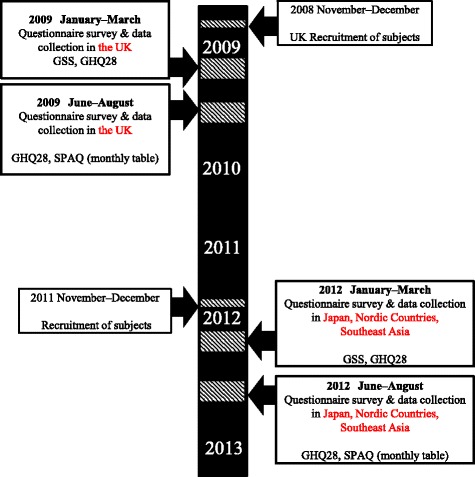
Timing of the surveys. GSS: Global Seasonality Score; GHQ: Global Seasonality Score; SPAQ: Seasonal Pattern Assessment Questionnaire

There was no difference in latitudes of the participants residing within each country. Surveys (winter and summer) in the UK were conducted in 2009, while surveys in other regions were conducted in 2012 (Fig. 1). Participants were recruited through facilitators at Japanese companies and Japanese cram schools (*Gakushū juku*) that provide supplemental lessons to Japanese pupils who study abroad. In addition, through Japanese permanent resident associations, participants were recruited through advertisements placed on association noticeboards. Questionnaires were distributed and returned by post or e-mail for both survey periods (summer and winter). When data were first gathered, participants were told that they would need to complete the survey twice; therefore, only people who agreed to participate twice took part in the study. Participants were also asked indicate their ID number from the first survey to permit their data to be matched for the second survey. In the first year (2009), 260 participants in the UK were invited to complete a self-administered questionnaire in winter, and 105 agreed to complete the second questionnaire in the summer. In the second year (2012), 68 participants in Nordic countries, 89 participants in Southeast Asia, and 153 participants in Japan were invited to complete a self-administered questionnaire in winter, and 40 participants in Nordic countries, 51 participants in Southeast Asia, and 96 participants in Japan agreed to complete the second questionnaire in summer. The number of participants is shown in Table 2.

**Table 2:** The number of participants completing each survey

Survey		Winter (Response rate %)	Summer (Response rate %)	Both (Response rate %)
2009	UK	260	233	105 (15.98%)
2012	Nordic countries	68 (63.55%)	44 (41.12%)	40 (18.69%)
	Southeast Asia	89 (63.57%)	57 (51.18%)	51 (21.25%)
	Japan	153 (75.74%)	117 (57.92%)	96 (20.55%)

### Measures

A longitudinal design was used to examine participants’ mental health at two points—in summer and winter—measured by the GHQ28 [11] and CES-D [12]. In the GHQ28, participants are required to indicate the extent to which each symptom is true of themselves on a 4-point scale, ranging 0–3. Total scores range 0–84, with higher scores implying poorer mental health. The CES-D is a self-report screening test for depression and depressive disorders. Participants indicate the extent to which depressive symptoms are true of them on a 4-point scale, ranging from 0 to 3. Total scores range 0–60, with higher scores implying more severe depression. The GSS comprises six items, scored on a 5-point scale ranging 0–4 (0 = no seasonality, 1 = mild, 2 = moderate, 3 = marked seasonality, and 4 = extreme seasonality), with total scores ranging 0–24 [7]. Based on participants’ GSS scores, respondents were divided into low (˂8), moderate (8–10), and high (≥11) seasonality groups. Table 1 shows the number of participants in each seasonality group by region.

Further, seasonal fluctuations in mood and behaviour patterns were examined based on participants’ GSS scores according to geographical region. The 10-item subscale of the Seasonal Pattern Assessment Questionnaire (SPAQ) measures seasonal mood and behaviour patterns by month.

Respondents have to indicate the period—a single month, cluster of months, any particular grouping, or no particular month(s) that is extreme on a regular basis—when they: (a) feel best, (b) gain most weight, (c) socialize most, (d) sleep most, (e) eat most, (f) lose most weight, (g) socialize least, (h) feel worst, (i) eat least, and (j) sleep least [8]. Responses for (a) through (e) are scored 1, ‘no change’ responses are scored 0, and responses (f) through (j) are scored −1. The SPAQ covers topics such as seasonal patterns in mood and behaviour by month.

### Data analyses

To verify differences in vulnerability to the influence of seasonal change according to region, paired *t*-tests were conducted for summer and winter GHQ28 total and subscale scores and CES-D by geographical region of residence. To analyse differences in scores for mood, social activity, sleep length, appetite, and weight by month, a Kruskal-Wallis test was performed. To further examine differences between geographical regions, a Mann-Whitney U test was performed on pairs of regions. In addition, the percentages of participants who responded ‘in a bad mood’, ‘increased interpersonal behaviour’, and ‘decreased sleeping hours’ were calculated with cross tabulations. To verify differences between seasons in GSS scores with gender, age, occupation, marital status, family size/presence of children, and duration of stay in country, a repeated measures ANOVA was conducted. IBM SPSS version 21.0 for Windows was used for statistical analyses.

Participants’ demographic characteristics are shown in Table 1.

## Results

**Fig. 2:**
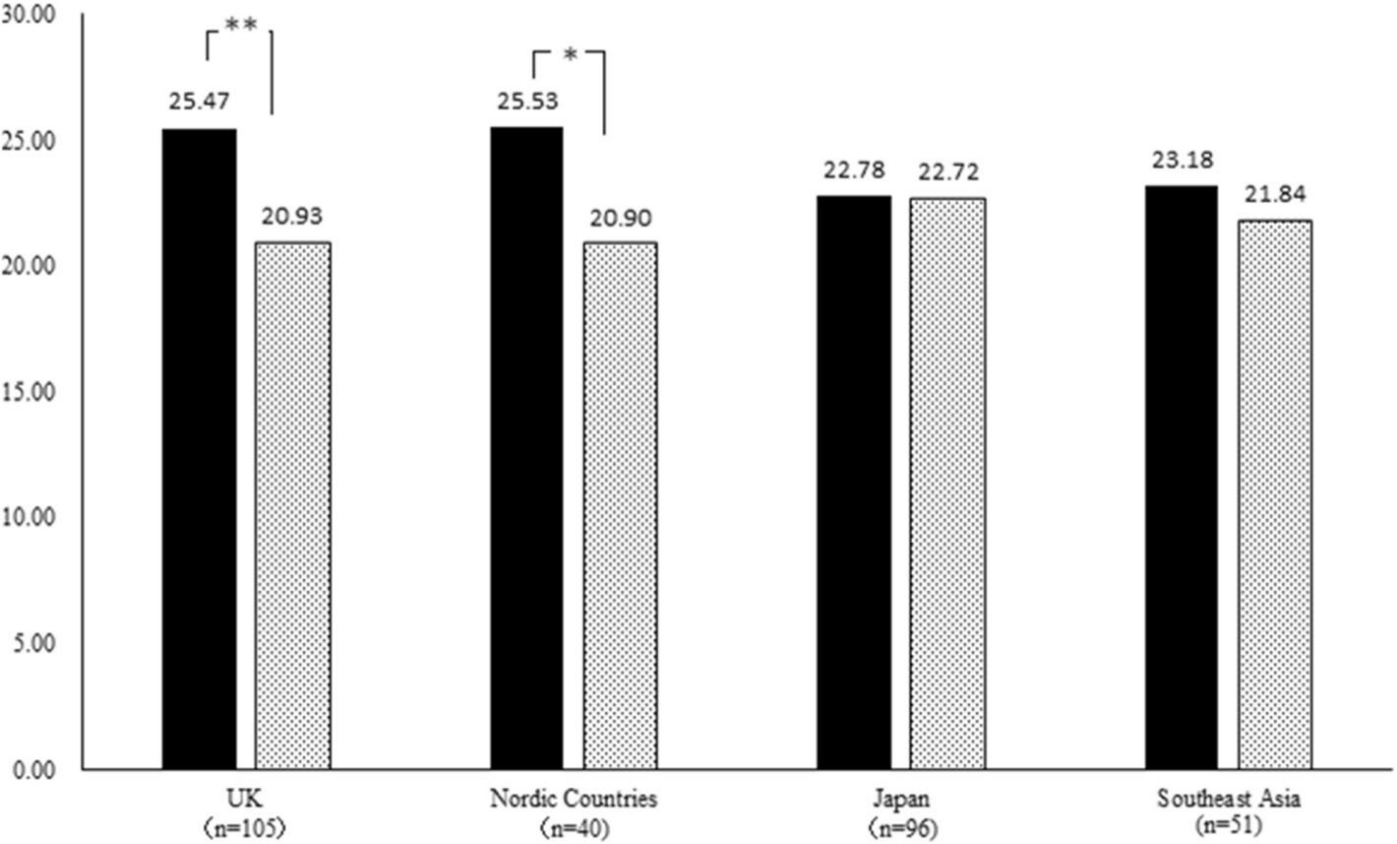
Results of the paired *t*-test on GHQ28 mean scores by season. Black shading: Winter; Dotted shading: Summer. Winter. Summer

As shown in Fig. 2, the paired *t*-test for GHQ28 scores by season demonstrated that total and subscale scores differed significantly by season for the UK (*t* = 4.08, *p* < .01) and Nordic countries (*t* = 2.30, *p* < .05) (Table 3). However, no significant seasonal differences in total or subscale scores among participants living in Japan and Southeast Asia were observed. In addition, the CES-D showed the same results such that the winter scores were significantly higher than the summer scores. The total score differed significantly by season for UK (*t* = 3.72, *p* < .01) and Nordic countries (*t* = 2.30, *p* < .05) (Table 3). This suggests that seasonal changes affected the mental health of Japanese migrants in high-latitude regions differently than they did for Japanese living in Japan or low-latitude regions.

**Table 3:** Differences by the paired *t*-test between summer and winter scores on the GHQ28 and CES-D

		UK (*n* = 105)				Nordic Countries (*n* = 40)				Japan (*n* = 96)				Southeast Asia (*n* = 51)			
		Mean	*SD*	*t*	*p*	Mean	*SD*	*t*	*p*	Mean	*SD*	*t*	*p*	Mean	*SD*	*t*	*p*
GHQ28 Total score	Winter	25.47	12.30	4.08	**	25.53	12.36	2.30	*	22.78	9.32	.00	*n.s.*	23.18	9.03	1.34	*n.s.*
	Summer	20.93	10.62			20.90	8.35			22.72	10.49			21.84	9.42		
Somatic symptoms (GHQ28)	Winter	7.45	4.16	2.57	**	6.90	3.84	1.47	*n.s.*	6.77	3.85	.22	*n.s.*	7.04	3.55	.41	*n.s.*
	Summer	6.29	4.08			6.08	3.25			6.66	3.61			6.84	4.09		
Anxiety/insomnia (GHQ28)	Winter	8.16	4.59	3.57	**	8.33	4.47	2.47	*	7.07	3.78	.41	*n.s.*	7.37	3.03	.11	*n.s.*
	Summer	6.63	3.99			6.60	3.20			6.9	3.77			7.33	3.60		
Social dysfunction (GHQ28)	Winter	7.31	2.79	2.57	**	7.30	2.48	2.40	*	6.76	2.15	.07	*n.s.*	6.43	1.80	1.13	*n.s.*
	Summer	6.48	2.70			6.03	2.28			6.73	2.52			6.12	1.93		
Severe depression (GHQ28)	Winter	2.54	4.20	2.79	**	3.00	4.24	1.25	*n.s.*	2.18	2.79	.73	*n.s.*	2.33	3.25	2.10	*n.s.*
	Summer	1.54	2.73			2.20	3.20			2.44	3.26			1.55	2.75		
CES-D	Winter	13.12	8.34	−3.72	**	13.55	8.84	2.30	*	12.21	6.99	.80	*n.s*	10.90	5.58	1.45	*n.s.*
	Summer	10.66	7.17			10.78	7.55			11.66	7.76			9.80	9.42		

*GHQ28* General Health Questionnaire 28, *CES-D* The Center for Epidemiologic Studies Depression Scale

*p < .05, **p < .01

In addition, 37.2% of participants in the UK obtained GSS scores of 8 or more (moderate to high seasonality), compared to only 25% of those in Japan (Table 1).

The results of the Kruskal-Wallis test revealed a significant difference in GSS scores between participants living in the UK and those living in Japan and other regions. Table 4 shows scores above/below 6 points on the GHQ28 by country. Table 5 shows the results of the Kruskal-Wallis test on GSS scores (*χ* ^2^ = 31.45, *p* < .0016). *T*-tests and ANOVAs were used to examine demographic data of UK participants who showed higher GSS scores. The results showed that female participants and younger participants tended to show higher GSS scores. Participants’ occupations did not significantly affect scores, but the duration of participants’ stays in the UK did. Participants who lived in the UK between 2 and 10 years showed higher GSS scores than participants who lived in the UK less than 2 years and more than 10 years (Table 6).

**Table 4:** Distribution of scores above/below 6 points on the GHQ28 by country

		UK (*n* = 105)				Japan (*n* = 96)			
		n	%	Mean	*SD*	n	%	Mean	*SD*
Winter	High Group (more than 6 points)	47	44.76	11.94	5.52	34	35.42	10.12	3.20
	Low Group (less than 6 points)	58	55.24	2.29	1.67	62	64.58	2.15	1.60
Summer	High Group (more than 6 points)	36	34.29	10.33	5.13	37	38.54	10.38	3.97
	Low Group (less than 6 points)	69	65.71	2.29	1.79	59	61.46	2.05	1.67
		Nordic countries (*n* = 40)				Southeast Asia (n = 51)			
		n	%	Mean	*SD*	n	%	Mean	*SD*
Winter	High Group (more than 6 points)	20	50.00	11.65	4.30	20	39.22	9.45	4.45
	Low Group (less than 6 points)	20	50.00	2.25	1.92	31	60.78	2.48	1.67
Summer	High Group (more than 6 points)	11	27.50	8.91	2.26	18	35.30	9.44	3.22
	Low Group (less than 6 points)	29	72.50	2.28	1.79	33	64.70	2.21	1.54

*GHQ28* General Health Questionnaire 28

**Table 5:** Results of the Kruskal-Wallis Test on GSS scores

	Median	Mean	*χ* ^2^	*p*-value	Multiple comparisons
UK (*n* = 105)	6.00	6.51	31.45	***	UK – Nordic countries *n.s.* UK > Southeast Asia *** UK > Japan ** Nordic countries > Southeast Asia *** Nordic countries – Japan *n.s.*
Nordic countries (*n* = 40)	5.00	5.60			
Southeast Asia (*n* = 51)	1.00	2.92			
Japan (*n* = 96)	4.00	4.76			

*GSS* Global Seasonality Score

** *p* < .0083, ***p < .0016 (after Bonferroni correction)

**Table 6:** Differences in GSS scores based on demographic data (UK)

Category		*n*	Mean	*SD*	*t* value/*F* value	*p* value	Multiple comparison
Gender	Male	49	5.31	4.29	−1.72	^†^	
	Female	211	6.52	4.46			
Age	20s	25	8.32	3.87	5.74	**	20s > 40s * 20s > 50s ** 30s > 40s * 30s > 50s **
	30s	73	7.39	4.77			
	40s	130	5.70	4.01			
	50 and over	32	4.58	4.02			
Occupation	Student	17	8.65	4.14	3.06	*	Others > Part-time^†^
	Housewife	114	6.23	4.31			
	Full-time	100	10.00	4.42			
	Part-time	7	4.78	5.51			
	(Other)	−18	6.35	4.35			
Family status	Unmarried	57	7.33	4.19	2.15	*	Unmarried > Married *
	Married	196	5.92	4.41			
	(Other)	(7)	8.00	6.33			
Children	Yes	163	6.20	4.69	-.42	n.s.	-
	No	97	6.43	4.02			
Duration of stay	Within 2 years	60	5.75	4.13	2.98	*	2–10 years > over 10 years †
	2–10 years	131	6.95	4.64			
	Over 10 years	69	5.51	4.20			

*GSS* Global Seasonality Score

^†^ *p* < .10, *p < .05, **p < .01

GHQ scores in Southeast Asia and Japan did not show seasonal differences with gender, age, occupation, marital status, and duration of stay in the country. GHQ scores in the UK showed a main effect of seasonal differences for gender, age, occupation, marital status, family size/presence of children, and duration of stay in the country. Only duration of stay in the country showed an interaction. GHQ scores in Nordic countries did not show meaningful differences by marital status nor were there any interactions with other factors (Table 7).

**Table 7:** Results of the repeated measures ANOVA for GHQ28 (UK and Nordic countries)

Main effect								Interaction effect					
			*n*	Mean	*SD*	*F*	*p-*value			Mean	*SD*	*F*	*p-value*
UK (*n* = 105)	Gender	Male	23	20.33	2.07	2.48	*n.s.*	Male	Winter	22.04	2.55	.27	*n.s.*
		Female	82	24.01	1.09				Summer	18.61	2.21		
	Season	Winter	105	24.24	1.44	9.40	**	Female	Winter	26.43	1.35		
		Summer		20.10	1.25				Summer	21.59	1.17		
	Age	39 years and under	40	24.94	1.57	1.98	*n.s.*	39 years and under	Winter	28.15	1.93	1.79	*n.s.*
		40 years and above	65	22.13	1.23				Summer	21.73	1.68		
	Season	Winter	105	25.98	1.22	18.42	**	40 years and above	Winter	23.82	1.51		
		Summer		21.09	1.07				Summer	20.45	1.32		
	Occupation	Full time	41	22.24	1.56	.62	*n.s.*	Full time	Winter	25.72	1.55	.65	*n.s.*
		Other	64	23.81	1.25				Summer	21.91	1.33		
	Season	Winter	105	25.4	1.24	17.2	**	Other	Winter	25.07	1.93		
		Summer		20.66	1.06				Summer	19.42	1.66		
	Family status	Unmarried	25	25.02	2.00	1.09	*n.s.*	Unmarried	Winter	27.92	2.46	.40	*n.s.*
		Married	80	22.63	1.12				Summer	22.12	2.13		
	Season	Winter	105	26.31	1.41	14.40	**	Married	Winter	24.7	1.37		
		Summer		21.34	1.22				Summer	20.56	1.19		
	Children	Yes	64	22.9	1.25	.15	*n.s.*	Yes	Winter	24.86	1.54	.47	*n.s.*
		No	41	23.67	1.57				Summer	20.94	1.33		
	Season	Winter	105	25.64	1.23	16.94	**	No	Winter	26.42	1.93		
		Summer		20.93	1.07				Summer	20.93	1.67		
	Duration of stay	90 months and under	68	24.46	1.20	3.16	^†^	90 months and under	Winter	27.56	1.46	4.21	*
		91 months and above	37	20.88	1.62				Summer	21.37	1.29		
	Season	Winter	105	24.59	1.23	11.21	**	91 months and above	Winter	21.62	1.98		
		Summer		20.75	1.09				Summer	20.14	1.75		
Nordic countries (*n* = 40)	Gender	Male	6	23.33	2.77	.00	*n.s.*	Male	Winter	24.33	5.11	.30	*n.s.*
		Female	34	23.19	1.87				Summer	22.33	3.44		
	Season	Winter	40	25.03	2.77	1.56	*n.s.*	Female	Winter	25.74	2.15		
		Summer		21.49	1.87				Summer	20.65	1.45		
	Age	39 years and under	24	22.63	1.73	.29	*n.s.*	39 years and under	Winter	24.83	2.55	.02	*n.s.*
		40 years and above	16	24.09	2.12				Summer	20.42	1.72		
	Season	Winter	40	25.7	2.02	5.06	*	40 years and above	Winter	26.56	3.12		
		Summer		21.02	1.36				Summer	21.63	2.11		
	Occupation	Full time	15	21.9	2.18	.58	*n.s.*	Full time	Winter	28.28	2.4	2.97	^†^
		Other	25	24.00	1.69				Summer	19.72	1.66		
	Season	Winter	40	24.61	1.95	2.97	^†^	Other	Winter	20.93	3.09		
		Summer		21.29	1.36				Summer	22.87	2.15		
	Family status	Unmarried	10	21.50	2.68	.55	*n.s.*	Unmarried	Winter	21.8	3.9	1.35	*n.s.*
		Married	30	23.78	1.54				Summer	21.2	2.67		
	Season	Winter	40	24.28	2.25	2.02	*n.s.*	Married	Winter	26.77	2.25		
		Summer		21	1.54				Summer	20.8	1.54		
	Children	Yes	17	23.17	2.07	.00	*n.s.*	Yes	Winter	24.41	3.01	2.83	*n.s.*
		No	23	23.24	1.78				Summer	18.94	2.01		
	Season	Winter	40	25.77	1.98	6.66	*	No	Winter	24.13	2.59		
		Summer		20.65	1.32				Summer	22.35	1.73		
	Duration of stay	90 months and under	26	23.21	1.67	.00	*n.s.*	90 months and under	Winter	25.65	2.46	.03	*n.s.*
		91 months and above	14	23.21	2.28				Summer	20.77	1.66		
	Season	Winter	40	25.47	2.08	.03	*n.s.*	91 months and above	Winter	25.47	1.4		
		Summer		20.96	1.4				Summer	20.96			

*GHQ28* General Health Questionnaire 28

**p < .01, *p < .05, ^†^ *p* < .10

**Fig. 3:**
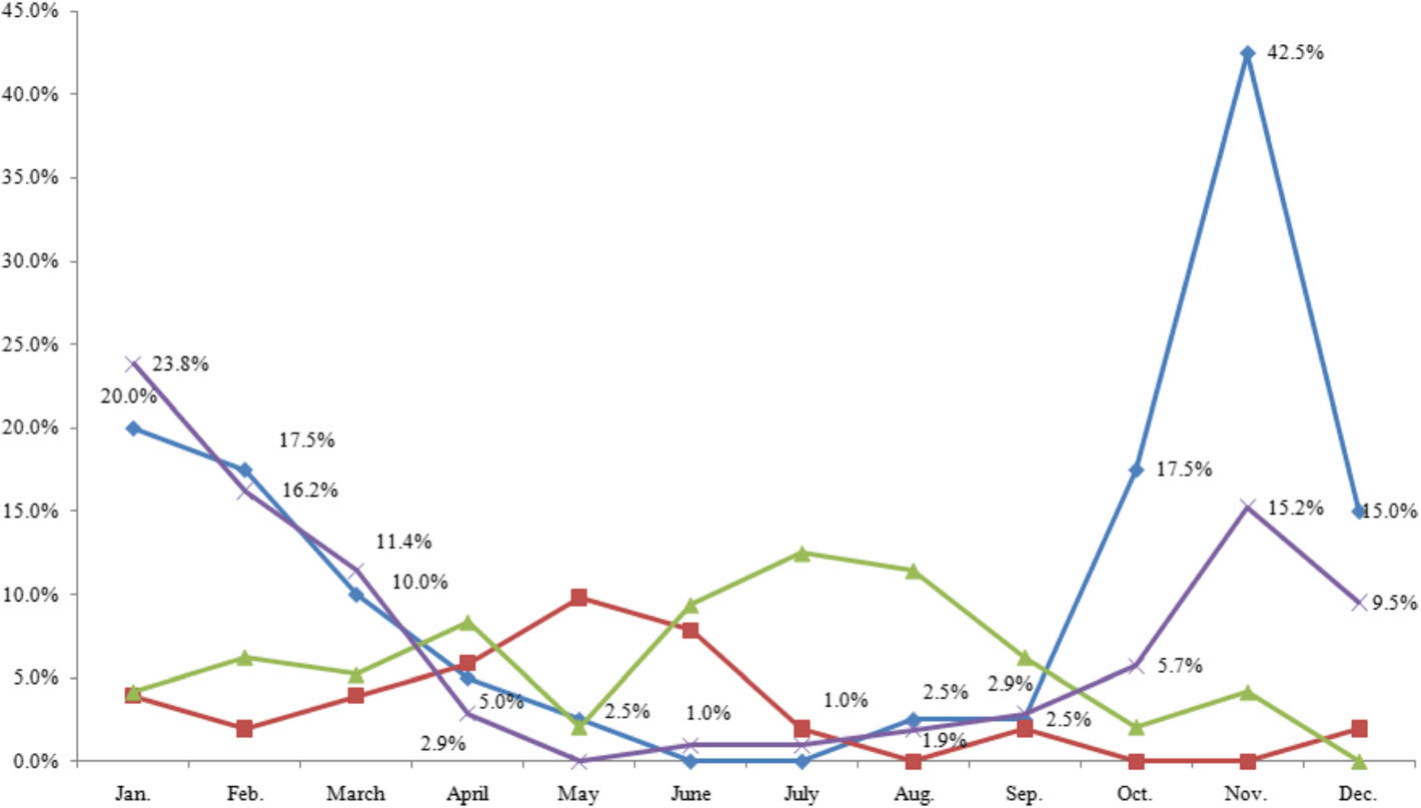
The monthly percentage of participants who responded ‘in a bad mood’ with cross-tabulations. Blue: Nordic countries (*n* = 40); Red: Southeast Asia (*n* = 51); Green: Japan (*n* = 96); Purple: UK (*n* = 105). Nordic countries (*n* = 40). Southeast Asia (*n* = 51). Japan (*n* = 96). U.K. (*n* = 105)

**Fig. 4:**
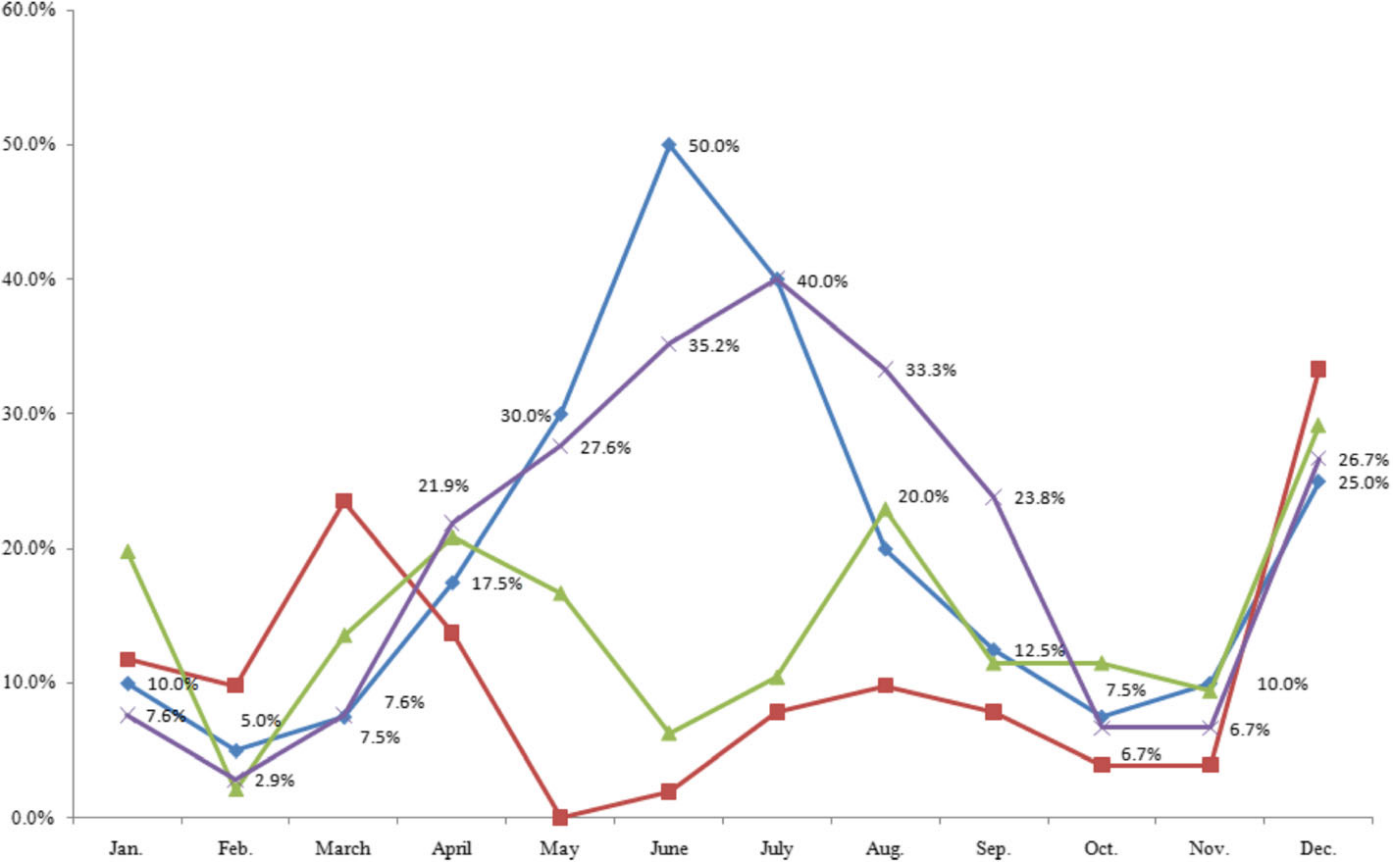
The monthly percentage of participants who responded ‘increase in interpersonal behavior’ with cross-tabulations. Blue: Nordic countries (*n* = 40); Red: Southeast Asia (*n* = 51); Green: Japan (*n* = 96); Purple: UK (*n* = 105). Nordic countries (*n* = 40). Southeast Asia (*n* = 51). Japan (*n* = 96). U.K. (*n* = 105)

**Fig. 5:**
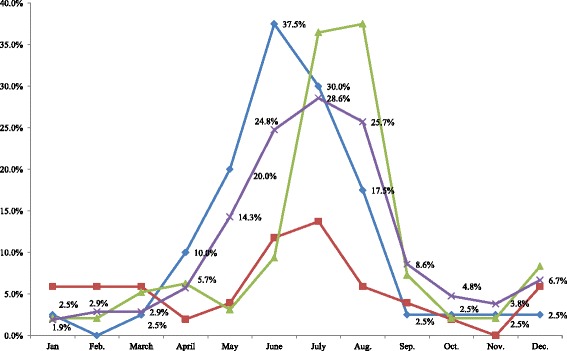
The monthly percentage of participants who responded ‘decreased sleeping hours’ with cross-tabulations. Blue: Nordic countries (*n* = 40); Red: Southeast Asia (*n* = 51); Green: Japan (*n* = 96); Purple: UK (*n* = 105). Nordic countries (*n* = 40). Southeast Asia (*n* = 51). Japan (*n* = 96). U.K. (*n* = 105)

Further, the results of the Kruskal-Wallis test revealed a significant difference between geographical regions with respect to seasonal changes in mood in winter and summer. The results suggest that unlike in Japan, lower mood in the UK and Nordic countries commences in October, and persists until January in the UK. We found a significant regional difference with respect to seasonal changes in interpersonal behaviour in winter and summer. According to the SPAQ results, residents in high-latitude regions (the UK and Nordic countries) tended to have a greater number of interpersonal interactions during summer and fewer interpersonal interactions during winter. Regarding the influence of seasonal change on length of sleep, the results revealed that during winter, participants in high-latitude regions had significantly longer sleep times compared to residents in Southeast Asia, while no significant differences were observed between participants in high-latitude regions and those in Japan (Tables 8 and 9). Collectively, these findings suggest that those living in high-latitude regions experience improvements in mental health during summer, as demonstrated by their improved mood and greater number of social interactions. Conversely, their mental health deteriorates during winter, as evidenced by worsened mood and lowered sociability. The analysis suggested that during summer time, Japanese living in higher latitude regions tend to have increased appetite and body weight compared to those living in lower latitudes. Such a difference was not found during winter. Thus, results suggested that the mood and interpersonal behaviour of people improved in summer and deteriorated in winter in countries of high latitudes (Figs. 3, 4 and 5).

**Table 8:** Analysis of mood and behaviour using the Kruskal-Wallis test for SPAQ scores

Mood				Interpersonal behavior			Sleep		
	*p*-value	Chi-squared value	Regional comparisons	*p*-value	Chi-squared value	Regional comparisons	*p*-value	Chi-squared value	Regional comparisons
January	**	16.18	UK < J, SA	**	21.7	UK < J, SA	*	14.62	SA < UK, J
February	*n.s.*	11.69	-	**	23.43	SA < UK, NC, J	**	10.36	SA < UK, J
March	*n.s.*	6.52	-	*n.s.*	10.88	E < SA	*n.s.*	1.97	–
April	*	11.71	SA < UK	*n.s.*	2.16	*–*	*n.s.*	2.98	–
May	**	50.79	J < UK; SA < UK, NC,J	**	25.23	SA < UK, NC, J	**	10.39	NC < J
June	**	97.01	J < UK,NC; SA < UK,NC	**	49.66	J < UK, NC; SA < UK, NC	*	14.77	UK, NC < J
July	**	104.48	J < UK,NC; SA < UK,NC	**	28.37	J < UK, NC; SA < UK, NC	*n.s.*	8.12	–
August	**	46.81	J < UK,NC; SA < UK,NC	*n.s.*	3.86	–	**	15.78	–
September	*n.s.*	11.35	–	*n.s.*	7.65	–	*n.s.*	6.2	–
October	**	21.24	UK, NC < J; SA < J	*n.s.*	3.24	–	*	12.72	J < NC
November	**	44.83	NC < UK; UK, NC < J; UK, N < SA	*n.s.*	9.6	J < UK	*n.s.*	7.71	–
December	*	13.97	UK < J,SA	*n.s.*	4.53	–	*n.s.*	5.66	–

*UK* United Kingdom, *NC* Nordic countries, *J* Japan, *SA* Southeast Asia

**p < .0016 *p < .0083 (Bonferroni correction)

**Table 9:** Analysis of mood and behaviour using the Kruskal-Wallis test for SPAQ scores

Weight				Appetite		
	*p*-value	Chi-squared value	Regional comparisons	*p*-value	Chi-squared value	Regional comparisons
January	**	22.20	UK < J; SA < J	*	13.52	SA < J
February	*n.s.*	3.01	–	*n.s.*	0.73	–
March	*n.s.*	1.82	–	*n.s.*	1.62	–
April	*	13.30	SA < UK	*n.s.*	3.11	–
May	*	11.94	SA < NC, J	**	15.48	SA < UK, NC, J
June	*n.s.*	3.71	–	*n.s.*	4.17	–
July	*n.s.*	4.87	–	**	22.43	J < UK, NC
August	*	12.02	UK < J	**	19.09	J < UK
September	*n.s.*	1.09	–	*n.s.*	5.89	–
October	*n.s.*	1.70	–	*n.s.*	11.07	–
November	*n.s.*	0.41	–	*n.s.*	11.07	–
December	*n.s.*	2.04		*n.s.*	1.57	–

*UK* United Kingdom, *NC* Nordic countries, *J* Japan, *SA* Southeast Asia

**p < .0016 *p < .0083 (Bonferroni correction)

## Discussion

Based on a study of the general population in Japan, Shirakawa et al. found that residents in high-latitude regions experienced greater fluctuations in mood, social life, sleep, and body weight compared to those in lower-latitude areas [7]. In the present study, although a seasonal (summer–winter) difference in GHQ28 scores among Japanese living in Japan and Southeast Asia (low-latitude regions) was not observed, a marked seasonal difference in scores among Japanese living in the UK and Nordic countries (high-latitude regions) was found. Duration of stay and season were shown to significantly interact. The general health of residents who had lived in the country for between 2 and 10 years was affected by season. In other words, individuals with shorter stays tended to be more affected by the seasons than those with longer stays. However, there was a tendency for mental health status to improve after individuals had sufficient time to adjust to life in the UK. Therefore, the mental health status during winter of people who are adjusting to life in the UK needs to be monitored carefully and support for these people should be offered. These results suggest that while Japanese in Tokyo are not significantly affected by seasonal change in terms of their mental health, those living in higher latitudes are susceptible to seasonal changes. Previous research indicates that women are more vulnerable to depression in winter compared to men [9, 13, 14]. Moreover, Suhail et al. showed that gender may be a major risk factor for low mood in winter [9]. Our study corroborates these findings: among Japanese residents in the UK, women were more susceptible to seasonal changes than were their male counterparts. Given that females were more susceptible to seasonal changes that might cause mental illness, support for women would be effective to prevent worsening of mental health during winter.

The analysis of fluctuations in mood and behaviour patterns of Japanese living in high-latitude regions over a 12-month period revealed fluctuations in mood and social activity, as well as decreased sleep during winter, followed by improvements in summer. These results suggest that seasonal changes have a substantial effect on mood and behaviour among Japanese living in high-latitude regions; they demonstrated better mood and had more social interactions in summer than in winter.

Activity is positively associated with mood. Thus, maintaining and promoting socializing opportunities during winter may help prevent depression in winter [15]. Behavioural activation therapy may effectively treat individuals with depression, as it teaches individuals to avoid rumination and restore stability for those with disrupted life patterns [16]. This study suggests that mental health interventions must necessarily consider seasonal changes, especially for individuals unaccustomed to them. Our findings on the relationship between seasonal fluctuations in mood and behaviour and high latitude are consistent with studies conducted in Japan [7, 17]. Although individuals in high-latitude regions experience declines in mood and behaviour in winter, they experience improvements in summer in the UK and in autumn in Japan. Additionally, while a decline in mood has been observed between the East Asian rainy season and summer in lower-latitude regions in Japan, in high-latitude regions, mood declined only during the winter and improved from early summer to early autumn.

Summer in Japan is perceived as an unpleasantly hot and humid season—with temperatures sometimes exceeding 30 °C—preceded by rain. In contrast, summer in high-latitude regions implies long daylight hours and pleasant weather. Thus, changes in mood and behaviour may also be related to environmental differences between regions/countries of residence. Shirakawa et al. found that seasonal fluctuations in mood were greater among residents in the high-latitude region in Japan compared to three other regions, and attributed this to substantial changes in climate [7]. This finding is consistent with the present study. Yet, the UK and Nordic countries are located at higher latitudes than Japan, and have different seasonal changes—shortened daylight hours and poor weather in winter and extended daylight and pleasant weather in summer—that may have yielded stronger correlations. Since the study period was 12 months, we could verify that mood and behaviour improved from winter to summer, but were unable to verify whether they decline from summer to winter. Future studies should examine this trend in detail, using comprehensive longitudinal data to clarify seasonal changes in mood and behaviour.

### Limitations

A limitation of the study was the skewed gender ratio (higher percentage of female compared to male respondents). Further, exploring whether differing lengths of residence and daily stressors—unlike those in the home country—influence these results would provide a more complex understanding of SAD, but was beyond the scope of this study. Finally, the finding regarding increased appetite and body weight requires attention, because these changes could have been atypical symptoms of depression, rather than an indication of improvement in mood.

## Conclusions

Compared to the domestic population, Japanese residents in high-latitude regions outside Japan experience significantly greater seasonal changes in mental health, mood, and behaviour, suggesting that their mental health issues need to be addressed. This involves understanding seasonal changes and encouraging the maintenance of social relationships during winter. The stressors in overseas countries are different from those in the home countries of migrants. Therefore, it is necessary to take these stressors into consideration when measuring the effectiveness of the support systems designed to help migrants in adapting themselves to seasonal changes in high-latitude regions. In high-latitude countries, weather conditions, especially during winter, tend to be poor and the duration of daylight is short. Therefore, measures to prevent lack of activity and to promote regular participation in events in these regions are needed.

## Abbreviations

CES-D: The Center for Epidemiologic Studies Depression Scale
GHQ28: General Health Questionnaire-28-Japanese version
GSS: Global Seasonality Score-Japanese version
SAD: Seasonal affective disorder
SPAQ: Seasonal Pattern Assessment Questionnaire
UK: United Kingdom
US: United States
